# Structure and Functions of Actin and Actin-Binding Proteins in *Leishmania*

**DOI:** 10.3390/pathogens14090948

**Published:** 2025-09-19

**Authors:** Chhitar M. Gupta, Saravanamuthu Thiyagarajan

**Affiliations:** Institute of Bioinformatics & Applied Biotechnology, Biotech Park, Electronic City, Phase I, Bengaluru 560100, India; sthiyaga@ibab.ac.in

**Keywords:** actin, actin-binding proteins, structure, functions, *Leishmania*

## Abstract

The actin cytoskeleton plays a crucial role in fundamental eukaryotic processes such as morphogenesis, motility, endocytosis, intracellular trafficking, and cell division. However, our understanding of actin and its associated proteins in trypanosomatid parasites like *Leishmania* remains limited. Over the past two decades, considerable progress has been made in elucidating the structure and functions of *Leishmania* actin and its core regulators. Notably, these findings are primarily derived from studies of the insect-stage promastigote form, while the roles of the actin machinery during the disease-causing amastigote stage within mammalian hosts remain largely unexplored. This review consolidates the current knowledge of actin and its interactors in *Leishmania* promastigotes, highlighting their potential roles in parasite development and stage-specific differentiation. Additionally, it explores the potential of targeting the cytoskeletal system as a strategy for novel therapeutic interventions against *Leishmaniasis*. The review concludes by identifying critical knowledge gaps and proposing future research directions to better understand actin-driven pathogenesis in this important human parasite.

## 1. Introduction

“Eukaryotic cells have evolved over billions of years, resulting in a wide variety of cell forms and functions. Despite this diversity, all eukaryotic cells share three fundamental characteristics: membrane-bound organelles, tightly regulated cytoskeletal networks, and complex signaling cascades” [1]. The eukaryotic cytoskeleton is composed of three principal elements: microtubules, microfilaments, and intermediate filaments. Actin, the primary protein constituent of microfilaments, exists in two physical states—monomeric (G-actin), predominantly ATP-bound in cells, and polymeric (F-actin), which is typically ADP-bound. The assembly and disassembly of filamentous actin, collectively termed actin dynamics, is a rapid and tightly regulated process that governs not only cell shape but also critical cellular functions, including motility [2], cytokinesis [3], intracellular trafficking [4], endocytosis [5], mitochondrial division [6], and autophagy [7].Beyond its cytoplasmic roles, actin also participates in key nuclear processes, such as DNA repair [8], chromatin remodeling [9], and transcriptional regulation [10]. Thus, actin filaments (F-actin) are indispensable for both structural integrity and functional versatility in eukaryotic cells.

Actin filaments (F-actin) are indispensable structural components of eukaryotic cells. They serve as scaffolds for myosin motor proteins and harness the energy of actin polymerization to generate force for driving various cellular processes [11]. The dynamics of actin microfilaments are tightly regulated by a diverse array of actin-binding proteins (ABPs) [12,13], whose activities are themselves governed by specific signaling pathways [14].

**Figure 1:**
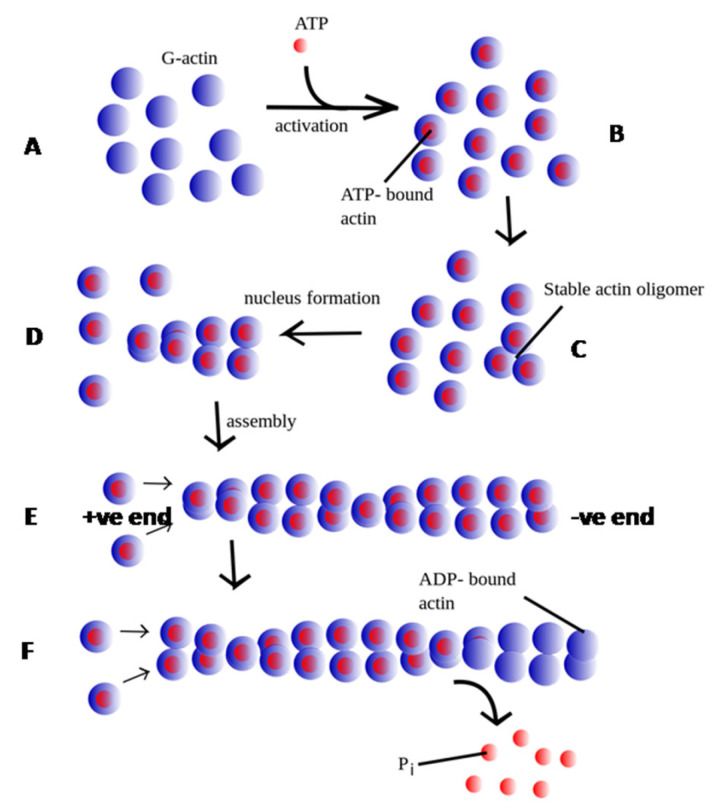
Schematic representation of actin polymerization and depolymerization dynamics. The process initiates with ATP binding to globular actin (G-actin; depicted as blue circles, **A**), represented by the attachment of small pink circles to individual G-actin monomers (**B**). Subsequently, a nucleation event occurs, characterized by the formation of a trimer of ATP-bound G-actin monomers (**C**). This nucleation step is energetically unfavorable in isolation but is stabilized through the intervention of nucleating proteins. Polymer elongation (**D**) proceeds with the addition of ATP-actin monomers to the rapidly polymerizing barbed (+) end (**E**) of the filament. Following incorporation, the ATP bound to G-actin undergoes hydrolysis to ADP and inorganic phosphate (Pi). This hydrolysis diminishes filament stability. Over time, Pi is released, leading to filament aging, which is followed by the dissociation of ADP-actin monomers (**F**). (Image adapted from [18]).

**Figure 2:**
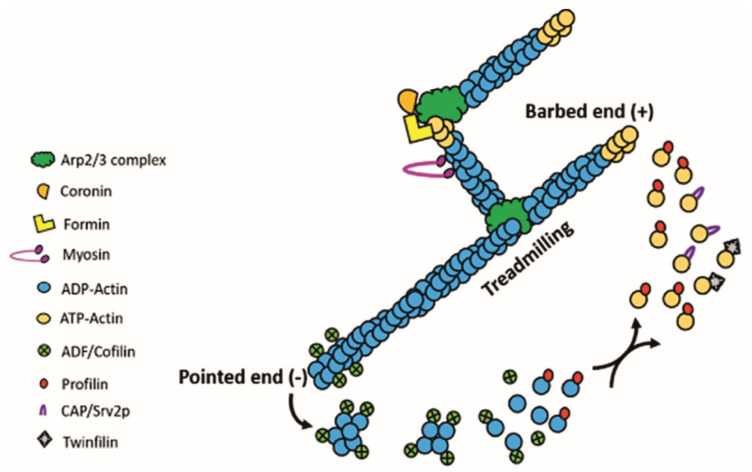
Schematic illustration of actin treadmilling. The diagram demonstrates that the dynamics of actin treadmilling are regulated by ADF/cofilins and profilin, which respectively promote filament disassembly and monomer availability, thereby modulating filament length. Additionally, the Arp2/3 complex nucleates new actin filaments by binding to actin monomers and existing filament sides, facilitating branched network formation. Formins, on the other hand, nucleate linear actin filaments through direct binding to actin monomers and cooperation with profilin. (The image is original artwork by [19]).

The assembly and disassembly of filamentous actin (Figure 1) proceed through three major steps: (1) Nucleation: The formation of a stable nucleus consisting of three actin monomers. Because this step is energetically unfavorable, cells employ nucleators such as formins and the Arp2/3 complex to initiate polymerization. Formins stabilize actin monomers to create a nucleus and, with the help of actin–profilin complexes, promote the post-nucleation growth of linear filaments [15]. In contrast, the Arp2/3 complex initiates the growth of branched filaments from the sides of pre-existing filaments [16]. (2) Elongation: Filaments elongate rapidly through the addition of ATP-bound G-actin to the polar (barbed) end. This process is accelerated by ABPs that locally increase the concentration of actin monomers. Formins, for example, remain associated with the growing barbed end, recruiting profilin-bound actin to sustain growth [15]. Filament length is regulated by capping proteins such as CAP_Z_, which prevents further elongation at the barbed end. (3) Disassembly: The ATP bound to G-actin in the growing filament undergoes hydrolysis, generating ADP and phosphate. As the filament ages, the phosphate group is released, leading to the dissociation of ADP-actin from the pointed end, a process catalyzed by actin depolymerizing proteins, such as ADF/cofilin [17]. The released ADP-actin undergoes exchange of bound ADP for ATP, allowing ATP-actin to enter a new round of polymerization. In a steady state, a dynamic equilibrium is achieved, where the length of actin filaments remains constant, with actin monomers continually associating with and dissociating from the ends. This phenomenon is commonly referred to as ‘actin treadmilling’ (Figure 2).

**Figure 3:**
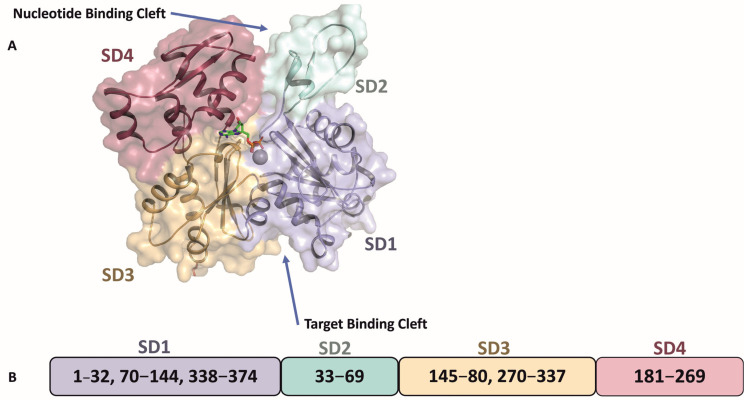
Schematic representation of the domain organization of the actin monomer. (**A**) The globular actin (G-actin) monomer comprises two major domains: a large domain and a small domain, each subdivided into two subdomains. The large domain consists of subdomains 1 (SD1) and 3 (SD3), while the small domain includes subdomains 2 (SD2) and 4 (SD4). Two prominent clefts are formed between SD2 and SD4, and between SD1 and SD3. The cleft between SD2 and SD4 corresponds to the nucleotide-binding site, where ATP binds (shown as sticks - colored by atom, carbon-green, nitrogen-blue, oxygen-red and phosphorous-orange) and the coordinated calcium ion as grey sphere, whereas the cleft between SD1 and SD3 serves as the target-binding site for actin-interacting binding proteins (ABPs). (**B**) A linear schematic illustrating the domain organization of actin, highlighting the specific amino acid residues that constitute each subdomain.

Holmes and colleagues were the first to report the crystal structure of G-actin complexed with DNase I [20]. This was followed by several studies on the crystal structures of actin with or without bound ligands reviewed in ref [21], revealing that the conformation of the actin monomer is fundamentally consistent. The 375 amino acid long actin polypeptide folds into two main domains: a large domain and a small domain, each containing two sub-domains. The small domain consists of subdomain 1 (SD1) and subdomain 2 (SD2), while the large domain contains subdomain 3 (SD3) and subdomain 4 (SD4). Between these subdomains, two clefts are formed: the ‘nucleotide binding cleft’, which binds ATP or ADP with a divalent cation such as Mg^2+^, and the ‘target binding cleft’, which is hydrophobic and delineates the region where most ABPs bind (Figure 3).

F-actin forms a double-stranded, staggered helix with a right-handed twist [21]. Its assembly is mediated by interactions between subdomain 2 (SD2) and subdomain 4 (SD4) of one G-actin protomer and subdomain 1 (SD1) and subdomain 3 (SD3) of another. Part of the amino acid sequence from SD2 involved in this process constitutes the DNase I binding site. Within the filament, most contacts occur between protomers of the same helical strand, where the hydrophobic DNase I binding loop (D-loop) inserts into a hydrophobic cavity formed between SD1 and SD3 of the adjacent protomer. Additional intra-strand interactions take place between SD4 and SD3. Inter-strand interactions are comparatively infrequent, the most prominent being between the hydrophobic plug of one protomer and the D-loop of its neighbor.

Actin is indispensable for cell survival and stands as the most conserved protein across all eukaryotic life, showing remarkable amino acid sequence identity among diverse lineages. For example, yeast and human actins share over 90% sequence identity [1]. In contrast, certain eukaryotes such as the intestinal parasite *Giardia lamblia* possess a single, highly divergent actin variant [22] that shares only ~59% identity with canonical actins, and their genome lacks genes for known actin-regulatory proteins [22]. Despite this divergence, these organisms employ actin for fundamental cellular processes, including morphogenesis, intracellular trafficking, and cytokinesis, much like other eukaryotes [23]. Similarly, other unicellular eukaryotic pathogens, including *Plasmodium*, *Toxoplasma*, *Trypanosoma*, *Leishmania*, and *amoebae* harbor actins with unusual properties, and some of their associated regulatory proteins exhibit novel architecture and unique cellular functions [24]. This review synthesizes current knowledge on the structure and functions of actin and actin-binding proteins in human-pathogenic Trypanosomatidae, with a focus on *Leishmania* species. 

## 2. *Leishmania* Actin

**Figure 4:**
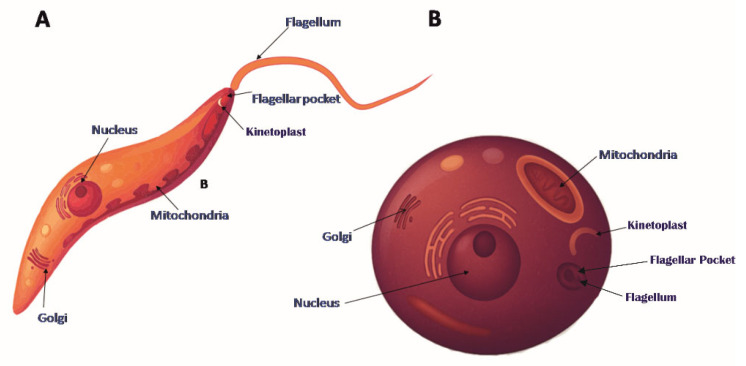
Schematic representation of *Leishmania* life cycle stages. (**A**) Promastigote and (**B**) amastigote forms. The base image was obtained from Shutterstock (https://www.shutterstock.com/, accessed on 1 August 2025) and modified using AI-based tools.

Protozoan parasites of the *Leishmania* genus (classified in detail in Gupta et al., 2020 [19]) are responsible for a spectrum of human diseases, ranging from mild cutaneous lesions to severe and potentially fatal infections. Species such as *L. major*, *L. panamensis*, and *L. tropica* typically cause cutaneous *Leishmaniasis*, while *L. braziliensis* can lead to disfiguring mucocutaneous disease. The most severe form, visceral *Leishmaniasis*, is caused by *L. donovani*, *L. infantum*, and *L. chagasi*, and can be fatal if left untreated. Globally, *Leishmania* infections affect millions, with an estimated 700,000 to 1 million new cases reported each year and 20,000 to 40,000 associated deaths [25]. These parasites have a digenetic life cycle involving two distinct morphological forms: the flagellated, spindle-shaped and motile promastigotes, which inhabit the gut of sandfly vectors, and the oval-shaped, non-motile amastigotes (Figure 4), which reside within mammalian macrophages [26]. Notably, unlike higher eukaryotes, *Leishmania* species exhibit a highly reduced actin cytoskeleton.

Although early studies failed to detect actin protein in *Leishmania* due to its inability to bind DNase I, lack of phalloidin staining, and absence of visible filaments under electron microscopy [27,28], genomic analyses later confirmed the presence of a single, highly divergent actin gene (~70% identity to mammalian actin) (Table 1) and a set of ten actin-binding proteins (ABPs) [29]. These proteins collaborate to regulate actin filament nucleation, polymerization, and disassembly, ensuring the proper functioning of actin remodeling-driven cellular processes. However, the absence of certain regulatory proteins, such as Actin Interacting Proteins (Aip1), which accelerates filament severing and pointed end depolymerization [30], and Capping Protein (CAPz), which controls filament barbed end polymerization [31], indicates that actin dynamics are regulated differently in *Leishmania* parasites compared to higher eukaryotes.

The presence and intracellular distribution of actin in *L. donovani* promastigotes was first reported by Sahasrabuddhe et al. (2004) [32], using antibodies raised against recombinant *L. donovani* actin (LdAct). Actin was found to be abundantly expressed in both the promastigote and amastigote stages [32]. In promastigotes, it localized to several cellular compartments, including the flagellum, flagellar pocket, nucleus, and kinetoplast, as well as to the nuclear, vacuolar, and cytoplasmic sides of the plasma membrane. Especially, actin associated with the plasma membrane was found to co-localize with subpellicular microtubules, while kinetoplast-associated actin co-localized with the kDNA network. These observations suggest potential roles for actin in regulating cell shape, endocytosis, and functions related to the kinetoplast and nucleus.

Unlike in mammalian cells, where actin typically forms long filaments, *Leishmania* actin primarily exists as granules and patches. These structures cannot be visualized using fluorescently labeled phalloidin and are not disrupted by cytochalasin D [32]. This finding was further validated through in vitro studies using purified recombinant LdAct [33]. Recombinant LdAct exhibited marked differences from canonical actins: it neither bound to nor inhibited DNase I, and its polymerized form did not interact with actin-stabilizing toxins (e.g., phalloidin) or actin-disrupting toxins (e.g., cytochalasins). Additionally, during polymerization, LdAct showed significantly higher ATPase activity than either mammalian or yeast actins, indicating that LdAct filaments are intrinsically more dynamic [33].

Of particular interest is the localization of LdAct to the kinetoplast [32], a unique finding, as no other eukaryotic cell has been shown to localize actin within the mitochondrion. This observation was reinforced by in vitro experiments demonstrating ability of LdAct to bind and nick kDNA, converting catenated minicircles into open forms [34]. This kDNA-nicking activity depended on both the polymerized state of LdAct and ATP hydrolysis. Structural analysis indicated that DNA binding is primarily mediated through electrostatic interactions involving the highly divergent DNase I–binding loop of LdAct and the DNA major groove [34]. In addition to binding DNA, LdAct was shown to interact with bacterial type II topoisomerase, inhibiting its decatenation activity. These findings suggest a potential role for actin in kDNA remodeling [35] within *Leishmania* parasites, although further studies are needed to clarify its precise mechanistic function.

Like rabbit muscle actin, purified LdAct polymerized optimally in the presence of Mg^2+^ and ATP. However, unlike most actins, LdAct was also capable of polymerizing in the absence of Mg^2+^, though it failed to polymerize in the presence of Mg^2+^ alone or with GTP [33]. Interestingly, His_6_-tagged LdAct polymerized only within a narrow pH range and predominantly formed bundles rather than filaments [33]. In contrast, untagged *L. major* actin (LmAct) formed stable filaments under standard polymerization conditions used for other actins [36], suggesting that the N-terminal His_6_ tag in LdAct may have influenced its polymerization behavior.

**Figure 5:**
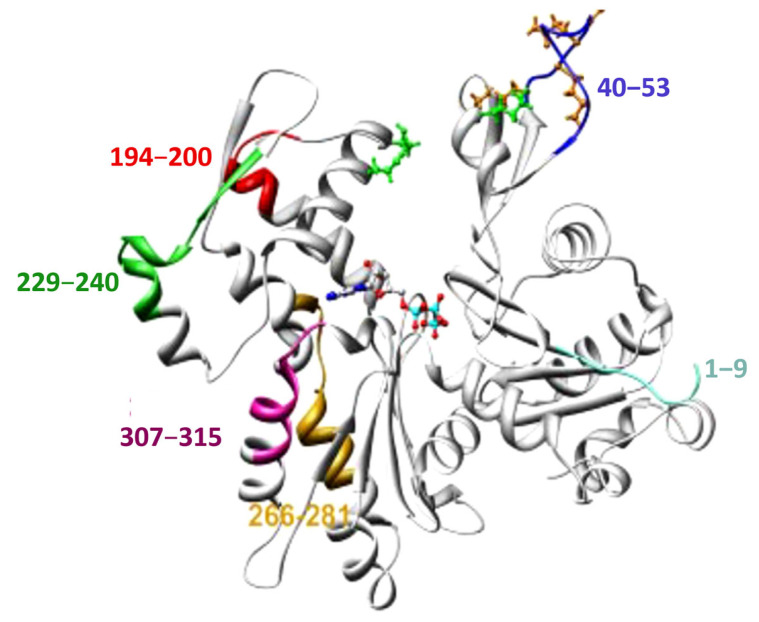
Average molecular dynamics–simulated homology model of *Leishmania donovani* actin (LdAct). Divergent amino acid stretches are highlighted in color: residues 1–9 (subdomain 1), 40–53 (subdomain 2), 266–281 and 307–315 (subdomain 3), and 194–200 and 229–240 (subdomain 4). Divergent residues within the DNase-I binding loop are shown as brown ball-and-stick representations; these positions correspond to substitutions in LdAct that form strong interactions with DNase-I in the actin–DNase-I complex crystal structure. Conserved residues that mediate weak interactions with DNase-I are shown as green ball-and-stick representations.

Computational and molecular modeling studies have shown that LdAct retains the conserved core structure of canonical actins, comprising four subdomains (I–IV) arranged in a characteristic globular fold [33]. Despite this structural conservation, LdAct displays substantial sequence divergence from mammalian actins, particularly in residues 1–9, 40–53, 194–200, 229–240, 266–281, and 307–315, most of which are surface-exposed in mammalian actins (Figure 5). These divergent regions are thought to alter surface charge distribution, the ATP-binding cleft, Mg^2^-binding sites, and the hydrophobic loop (H-plug) involved in monomer–monomer interactions, thereby contributing to unique biochemical properties of LdAct [33].

**Figure 6:**
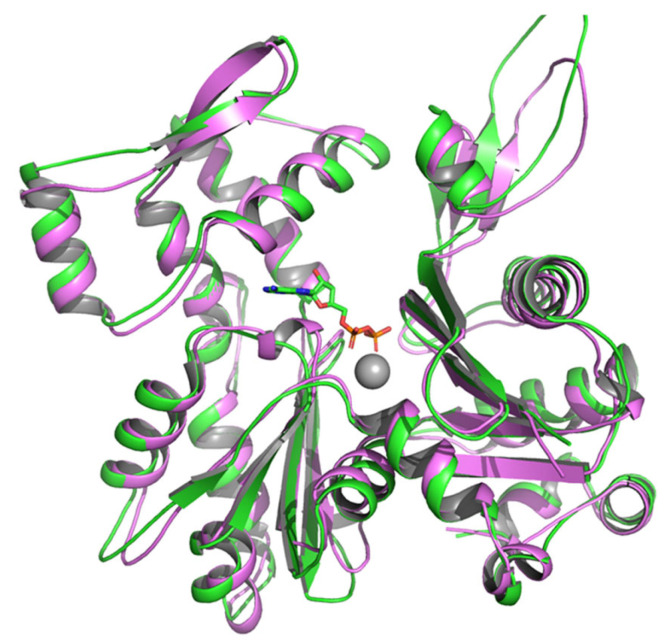
Structural comparison of *Leishmania* and human cytosolic actin. Superposition of human cytosolic actin (green cartoon, AlphaFold structure) and *Leishmania major* actin (magenta cartoon, PDB ID: 7Q8C). ATP bound to *L. major* actin is shown as sticks (colored by atom, carbon-green, nitrogen-blue, oxygen-red and phosphorous-orange), and the coordinated magnesium ion is depicted as a gray sphere. The root-mean-square deviation (RMSD) of common Cα atoms between the two structures is 1.3 Å.

High-resolution structural analyses using cryo-electron microscopy (cryo-EM) and X-ray crystallography have confirmed that LmAct retains the typical eukaryotic actin fold, composed of four subdomains (Figure 6) [36]. However, notable differences exist between *Leishmania* and mammalian actins, particularly in regions such as the D-loop, H-plug, and barbed-end cleft—elements known to play critical roles in filament stability. These structural differences likely underlie the distinct biochemical behavior observed in *Leishmania* actins [36].

## 3. *Leishmania* Actin-Binding Proteins (ABPs)

Unlike higher eukaryotes, lower eukaryotic organisms such as *Leishmania* parasites possess a simplified actin cytoskeleton, reflecting their comparatively limited functional requirements. Consequently, these organisms express only a restricted set of actin-regulatory proteins. This includes actin nucleating and elongation factors (two formin isoforms and a single Arp2/3 complex), actin monomer-binding proteins (one copy each of profilin, twinfilin, and CAP), F-actin-binding proteins (one copy of coronin and two isoforms of myosin), and F-actin depolymerizing/severing proteins (a single copy of ADF/cofilin). All other core actin-binding proteins typically found in higher eukaryotes (as listed in Table 2) are entirely absent from the *Leishmania* genome [29]. The structural characteristics and functional roles of these proteins in regulating actin cytoskeleton dynamics and organization in *Leishmania* are summarized below.

### 3.1. The Arp2/3 Complex

The Arp2/3 complex (Actin-Related Protein 2/3 complex) is a highly conserved, seven-subunit protein complex that plays a central role in actin polymerization and cytoskeletal organization in eukaryotic cells [37,38]. It is composed of two actin-related proteins, Arp2 and Arp3, along with five additional subunits, ARPC1 through ARPC5. Arp2 and Arp3 share structural similarity with conventional actin and are directly responsible for initiating the formation of new actin filaments, whereas the ARPC1–5 subunits stabilize the complex and modulate its activity [37].

The primary function of the Arp2/3 complex is to nucleate branched actin filament networks. It achieves this by binding to the side of a pre-existing (mother) filament and initiating the growth of a new (daughter) filament at a characteristic angle of ~70°, resulting in a branched network [37]. Such architecture is essential for generating the dynamic filament arrays required for diverse cellular processes, including endocytosis, cell migration, and morphogenesis.

Bioinformatic and comparative genomic analyses [39] suggest that orthologs of all seven canonical Arp2/3 subunits are likely encoded in the *Leishmania* genome. However, only five subunits have been confidently identified via sequence homology. The remaining two, ARPC1 and ARPC5, are not readily detectable, probably due to substantial sequence divergence that obscures their identification using standard homology-based methods. To date, there is no direct experimental confirmation of the composition or activity of the Arp2/3 complex in *Leishmania*. Nevertheless, functional roles analogous to those in mammalian and yeast systems have been proposed, particularly in driving the membrane deformation events required for endocytosis. In this context, the complex is thought to generate the mechanical force needed for plasma membrane invagination and endocytic vesicle formation [40].

The activity of the Arp2/3 complex is tightly regulated by upstream modulators, notably nucleation-promoting factors (NPFs) such as WASP (Wiskott–Aldrich Syndrome Protein) and SCAR/WAVE, which activate the complex and enhance its nucleation activity [41]. Conversely, inhibitory proteins like coronin suppress Arp2/3 activity to prevent excessive or aberrant filament formation [42,43]. Interestingly, *Leishmania* species do not appear to encode a clear WASP ortholog. However, they do express a protein with weak sequence homology to WASH-1, a component of the WASH complex known to regulate Arp2/3 activity in other eukaryotes [29].

### 3.2. Formins

Formins are typically long, multidomain proteins characterized by both conserved and variable regulatory regions. Their defining structural elements are the formin homology 1 (FH1) and formin homology 2 (FH2) domains [44]. Formins function as dimers, with each monomer contributing to actin filament binding and regulation. Members of the formin superfamily display diverse nucleation and elongation capabilities, enabling the assembly of a wide range of actin-based structures [44].

At the core of formin-mediated actin nucleation is the FH2 domain, which forms a donut-shaped homodimer that associates with the barbed end of actin filaments [45]. The FH2 domain alone is sufficient to nucleate actin filaments and remain processively attached to their growing barbed ends. It is thought to stabilize transient actin dimers or trimers, thereby promoting filament assembly [45]. Located upstream of FH2, the FH1 domain contains polyproline stretches that bind profilin–actin complexes. This interaction facilitates the local enrichment of actin monomers at the barbed end, thereby accelerating filament elongation [46,47].

In *Leishmania*, however, profilin appears to inhibit the role of formins in actin nucleation and elongation [40], suggesting a divergence from the regulatory mechanisms observed in other eukaryotes. As a result, further studies are needed to fully elucidate the structure and function of *Leishmania* formins, which may reveal unique aspects of cytoskeletal regulation in trypanosomatids. Particularly, the only study conducted so far indicates that *Leishmania* formins function primarily as actin-bundling proteins rather than as classical nucleators or elongators [39].

### 3.3. Profilin

Profilin is a small, evolutionarily conserved protein that plays a central role in regulating actin dynamics. It binds actin monomers and facilitates their incorporation into growing actin filaments [48,49]. Initially identified as G-actin–sequestering proteins, profilins are now recognized as essential regulators of the actin cytoskeleton. They interact with a variety of actin-binding proteins and link membrane lipids to the cytoskeleton [49]. Functionally, profilins inhibit spontaneous nucleation of actin filaments, promote elongation at filament barbed ends, and block monomer addition at pointed ends [48]. Through these mechanisms, profilin modulates actin polymerization, contributing to cytoskeletal organization and remodeling, which are vital for cell shape, motility, and intracellular transport [49,50].

**Figure 7:**
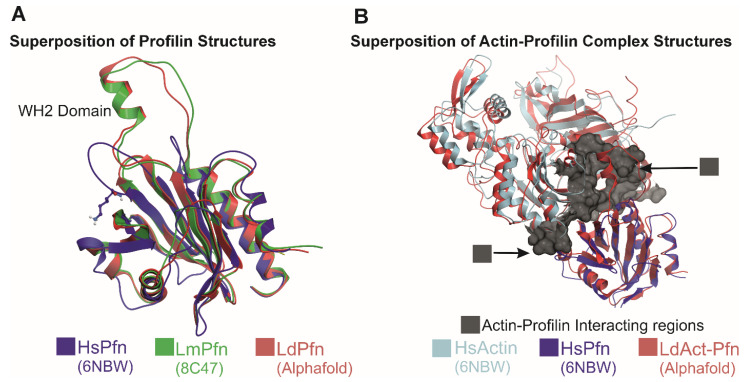
Structural comparison of profilin and the actin–profilin complex in humans and *Leishmania*. (**A**) Cartoon representation of the superimposed profilin structures: human profilin (HsPfn, blue; PDB ID: 6NBW, chain C), *Leishmania major* profilin (LmPfn, green; PDB ID: 8C47, chain A), and *Leishmania donovani* profilin (LdPfn, red; AlphaFold model). (**B**) Structural superposition of actin–profilin complexes from *L. donovani* (red; AlphaFold model) and human (cyan-blue; PDB ID: 6NBW). The actin region interacting with profilin is depicted as a gray surface. Region (1) highlights the HsAct–HsPfn interaction, whereas region (2) denotes the corresponding interaction in *Leishmania*. The figure represents original artwork by the authors, illustrating differences in profilin–actin binding between mammalian and *Leishmania* systems.

Profilin is highly conserved across eukaryotes, including in *Leishmania*, and typically consists of a single compact globular domain [51]. Like profilins in higher eukaryotes [49], *Leishmania* profilin possesses distinct binding sites for actin monomers, poly-L-proline (PLP) sequences, and phosphatidylinositol lipids [52]. It also catalyzes the exchange of ADP for ATP on actin monomers, enhancing their readiness for polymerization [52]. However, unlike most profilins, *Leishmania* profilins feature a unique α-helical insertion that engages the actin monomer’s target binding cleft (Figure 7) [40]. This insertion, conserved within the *Trypanosomatidae* family, resembles the WASP homology-2 (WH2) domain, a known actin-binding motif present in many cytoskeletal regulators [53]. The WH2-like motif enhances actin binding and accelerates nucleotide exchange, thereby promoting actin polymerization [40].

Beyond actin binding, profilin also interacts with PLP-rich proteins such as formins and Ena/VASP, which are involved in filament nucleation and elongation [49,50]. Formins contain FH1 and FH2 domains; the FH1 domain is rich in polyproline sequences that recruit profilin–actin complexes, delivering them to the FH2 domain for filament elongation. In most eukaryotes, profilin and formins synergize to enhance actin polymerization. However, *L. major* profilin (LmPfn) exhibits a distinct mechanism: it binds the barbed-end surface of actin, interacts with polyproline motifs, and inhibits spontaneous filament nucleation mediated by formins [40]. Additionally, profilin contains specific residues that mediate binding to microtubules [54].

In *L. donovani* promastigotes, profilin (LdPfn) is broadly distributed throughout the cell, including the flagellum, nucleus, and kinetoplast [52]. It regulates key processes such as endocytosis and intracellular trafficking [52]. LdPfn also interacts with various cellular proteins, including mitochondrial outer membrane porin, translation initiation factors like eIF4A1, and calpain-like cysteine proteases [55,56], implicating it in mitochondrial function [57], gene expression [58], parasite virulence [59,60], and possibly immune evasion [59,60]. Moreover, LdPfn contributes to G1-to-S phase progression and proper mitotic spindle orientation during cell division [55], potentially by regulating the expression of kinesin 13.1 [61], as suggested by transcriptomic comparisons between wild-type and heterozygous *LdPfn* mutants [55].

### 3.4. ADF/Cofilin

ADF/cofilins are key regulators of actin cytoskeleton dynamics, integrating signals from pH, phosphorylation, and lipid interactions to control filament severing and depolymerization [62,63,64]. They are essential for cellular motility, development, and disease processes [62,64]. Members of the ADF-H protein family, ADF/cofilins are small actin-binding proteins (13–19 kDa) [62]. In mammals, three isoforms exist: ADF (destrin), cofilin-1 (ubiquitous in non-muscle cells), and cofilin-2 (predominant in muscle) [63].

ADF/cofilins preferentially bind ADP–F-actin over ADP-Pi–F-actin or ATP–F-actin [62,63,64]. Binding is cooperative, with one molecule of ADF/cofilin per actin subunit at saturation, and induces a ~25° increase in filament helical twist [65,66]. This conformational change destabilizes inter-subunit contacts, rendering the filament mechanically unstable and prone to severing [65,66]. Severing generates more filament ends, boosting actin turnover [65].

Their activity is tightly regulated by pH and phosphorylation [67,68]. LIM kinases phosphorylate and inactivate ADF/cofilins, whereas slingshot phosphatases dephosphorylate and activate them [68]. Acidic conditions (pH 6.5–7.0) enhance depolymerization, while alkaline pH suppresses it. Interaction with phosphoinositides (e.g., PIP2) inhibits ADF/cofilin activity, and this inhibition is relieved at higher pH [69]. Their function is further modulated by proteins such as profilin, tropomyosin, and Aip1 [70,71,72].

**Figure 8:**
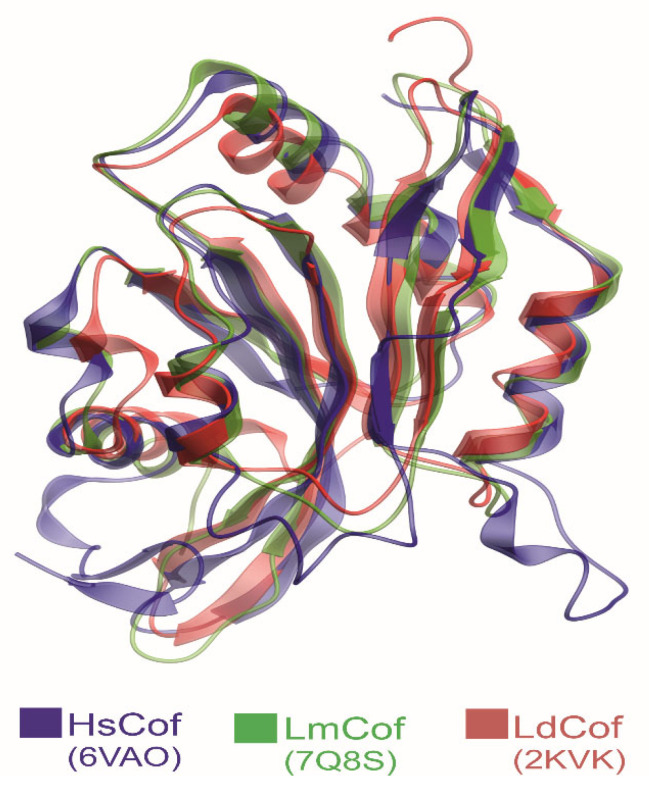
Structural comparison of human and *Leishmania* cofilins. Cartoon representation of the superimposed structures of human cofilin (HsCof, blue; PDB ID: 6VAO), *Leishmania major* cofilin (LmCof, green; PDB ID: 7Q8S, chain A), and *Leishmania donovani* cofilin (LdCof, red; PDB ID: 2KVK). The comparison highlights the high structural similarity between *Leishmania* and mammalian cofilins.

*Leishmania* parasites express a single ADF/cofilin homolog (LdCof) that possesses filament depolymerizing and severing activities, similarly to those found in other eukaryotes. Top of Form [73,74]. It localizes to the cell body, flagellum, and is highly concentrated in the flagellar pocket [73], the sole site for nutrient uptake [75,76], suggesting a role in endocytosis and vesicle trafficking. Structurally, LdCof resembles mammalian counterparts (Figure 8) [36,77], but with a few differences:

- An extended C-terminal α-helix critical for efficient severing [36].
- An isoleucine instead of serine at position 3, removing the canonical phosphorylation site [36].
- pH-independent depolymerizing activity [73].
- Absence of tropomyosin in the *Leishmania* genome [29], eliminating a key inhibitory mechanism seen in mammals [72].

Functionally, LdCof severs filaments >100-fold more efficiently than mammalian cofilins [36], enabling rapid actin turnover despite lacking Aip1. It depolymerizes filaments from both ends [36], supporting fast cytoskeletal remodeling necessary for endocytosis and other dynamic processes [36,73]. Reverse genetics studies show that LdCof-mediated actin remodeling regulates cell shape, motility, flagellum assembly, intracellular trafficking, and early cell division events, including basal body and kinetoplast separation, cleavage furrow progression, and flagellar pocket division [73,78].

### 3.5. Coronin

Coronins are a structurally diverse family of proteins characterized by conserved WD40 domains that form a β-propeller structure, along with a variable unique region and a C-terminal leucine zipper–type coiled-coil domain responsible for oligomerization [79,80,81]. They operate at the interface of cytoskeletal dynamics and cellular signaling, with their functions modulated by post-translational modifications and interactions with partner proteins such as cofilin and the Arp2/3 complex [82,83].

Coronins regulate actin dynamics by either inhibiting Arp2/3 complex–mediated nucleation or promoting cofilin-mediated filament disassembly [82]. Acting as scaffold, they coordinate actin remodeling during key cellular processes such as migration and vesicle trafficking [83]. Additionally, coronins interact with microtubule-associated proteins, thereby linking the actin and microtubule networks [84]. Their activity is regulated through phosphorylation by protein kinase C (PKC) and calcium/calmodulin signaling, which influence both their localization and function [85].

*Leishmania* expresses a single coronin homolog (LdCor), which retains the core structural features of mammalian coronins but contains a coiled-coil domain with five heptad repeats—significantly more than the 2–3 heptads found in most mammalian coronins [86]. This extended region enables LdCor to form higher-order oligomers, such as tetramers or pentamers [87]. Structural studies show that its coiled-coil domain assembles into an asymmetric, four-helix antiparallel bundle due to an axial shift in one of the helices [88].

LdCor binds actin filaments via its unique region [89]. However, deletion of the coiled-coil domain abolishes oligomerization and impairs actin filament assembly, indicating that the coiled-coil domain is essential for oligomerization but not for direct actin binding [89].

LdCor localizes to actin-rich regions in *Leishmania*, including the cell cortex and membrane protrusions, underscoring its role in cytoskeletal organization [86]. In addition to stabilizing actin filaments, LdCor remodels microtubules by interacting with the kinesin motor protein K39 during cytokinesis [90]. It also plays a critical role in basal body duplication and is essential for the survival of promastigotes in culture [90]. Bioinformatic analyses further predict its involvement in phagosome formation, highlighting its multifaceted functions in parasite biology [39].

### 3.6. Twinfilin

Twinfilin is a widely conserved and abundant actin monomer–binding protein, characterized by the presence of two actin-depolymerizing factor homology (ADF-H) domains [91], which enable interactions with both monomeric (G-actin) and filamentous (F-actin) forms of actin. Its C-terminal region often contains additional motifs that modulate its activity and subcellular localization [91]. Although twinfilin is structurally conserved across eukaryotic species, its functional roles exhibit species-specific adaptations, particularly in *Leishmania*.

Through specialized actin-binding sites, twinfilin associates with filament barbed ends, a key site for the regulation of actin filament turnover [91]. It sequesters G-actin, thereby limiting the addition of monomers to growing filaments and controlling the pool of polymerizable actin [92,93]. Its depolymerization activity is nucleotide-dependent: twinfilin promotes disassembly of newly formed filaments containing ADP–Pi, but slows the breakdown of older, ADP-bound filaments [94].

Early models described twinfilin as a classical barbed-end capping protein. However, it is now recognized that twinfilin does not cap filaments in the traditional sense. Instead, it binds to barbed ends already occupied by capping protein and facilitates uncapping, thereby initiating filament depolymerization [95]. Recent studies using microfluidics-assisted TIRF microscopy have further characterized twinfilin as a dynamic, multifunctional regulator of barbed ends. It transiently caps barbed ends, depolymerizes filaments in a non-processive manner, and works in concert with formins to displace capping proteins, enabling rapid, age-dependent turnover of actin filaments [96].

In *L. major*, twinfilin exhibits significant divergence from its mammalian counterparts. It can inhibit barbed-end polymerization and drive filament depolymerization even in the absence of capping protein, supporting the rapid actin turnover critical to parasite physiology [36]. In *L. donovani*, twinfilin localizes specifically to the nucleolus. During cell division, it relocates to the nucleus, where it plays a direct role in spindle elongation and DNA synthesis during karyokinesis, thus linking actin regulation to nuclear function and cell division [97].

### 3.7. Cyclase-Associated Protein (CAP)

Cyclase-associated proteins (CAPs) are a highly conserved family of actin-binding proteins found across a wide range of organisms, including yeast, flies, plants, and mammals [98]. These multifunctional proteins contain several structural domains and play dual roles in regulating actin dynamics [98]. The N-terminal region of CAP enhances cofilin-mediated severing of actin filaments, while the C-terminal region promotes the recycling of actin monomers [98].

The most conserved region of CAP is the CARP domain (CAP and X-linked retinitis pigmentosa 2), which binds ADP-bound G-actin and catalyzes the exchange of ADP for ATP, regenerating polymerization-competent actin monomers [99]. The CARP domain typically functions as a homodimer, with each subunit interacting with one actin monomer. This dimeric structure forms extensive contact surfaces with actin, reflecting a strong and specific interaction [99]. The CARP domain engages actin through two distinct interfaces, known as the primary and secondary binding sites [99].

In addition to recycling monomers, CAP contributes to actin filament disassembly by binding to the pointed ends of cofilin-decorated filaments, promoting rapid depolymerization [100,101]. Through this dual mechanism, facilitating both filament disassembly and nucleotide exchange, CAP plays a central role in maintaining a dynamic pool of ATP-actin monomers necessary for continuous cytoskeletal remodeling

Unlike its mammalian counterpart, *Leishmania* CAP lacks the N-terminal domain required for pointed-end filament depolymerization. Instead, *Leishmania* expresses a truncated CAP isoform composed primarily of the C-terminal region. This truncated form retains the ability to bind ADP-actin monomers and catalyze nucleotide exchange, thereby sustaining actin dynamics by ensuring a constant supply of assembly-competent ATP-actin monomers [36].

### 3.8. Myosins

Myosins are a diverse group of actin-based motor proteins involved in numerous cellular functions [102,103]. They generally comprise three main domains: the head, neck, and tail. The head domain, located at the N-terminus, is a highly conserved motor region responsible for binding filamentous actin and generating force through ATP hydrolysis. The neck domain acts as a lever arm, amplifying the force produced by the head. It also serves as an attachment site for myosin light chains, which act as regulatory subunits. The tail domain, which varies significantly among myosins, determines cargo specificity, subcellular localization, and oligomerization. This domain may include coiled-coil regions (as in myosin II for dimer formation), cargo-binding segments, or membrane-association motifs [104,105]. Variations in domain structure and amino acid sequences have led to the classification of over 30 distinct myosin classes across various organisms [106,107].

The genome of the *Leishmania* parasite encodes two myosins: myosin 1B and a kinetoplastid-specific myosin initially identified as myosin XXI [29], which, after phylogenetic analysis, was reclassified as myosin 13 [108]. However, only myosin 13 (LdMyo13) is expressed in both the promastigote and amastigote stages of the parasite’s life cycle, indicating its essential role throughout development [109].

Similarly to other myosins, LdMyo13 comprises three main domains: a conserved N-terminal motor domain, a neck domain that typically contains IQ motifs for calmodulin binding, and a C-terminal tail domain involved in cargo binding [109,110]. The motor domain is actin-activated and binds calmodulin, which is necessary for motility but not for ATPase activity [110]. While the neck region lacks perfect IQ motifs, it features an extended dimerization region following the converter domain and extending to the molecule’s C-terminal end [111]. *Leishmania*-specific calmodulin-like proteins bind near the converter in a calcium-dependent manner [111].

The C-terminal tail of LdMyo13 contains two ubiquitin-associated (UBA) domains, although their function remains unclear. The motor domain also binds lipids via the dimerization region, with lipid binding and dimerization being mutually exclusive [111]. When calmodulin is not bound, the motor exists as a dimer and is nonmotile; upon Ca^2+^-calmodulin binding, it becomes monomeric and targets distinct lipid compartments. Membrane association is driven by a phospholipid-binding phox homology (PX) domain, which overlaps with the converter region [111].

In *L. donovani* promastigotes, LdMyo13 is prominently localized at the proximal region of the flagellum, and associates with the paraflagellar rod (PFR) [109]. This flagellar localization is determined solely by the tail region of LdMyo13, with the UBA-like domains playing a critical role [109,112]. Expression levels of LdMyo13 vary throughout promastigote growth, being higher in the stationary phase compared to the early or mid-log phases [113].

LdMyo13 exists in two cellular forms: detergent-soluble and detergent-insoluble [109]. The detergent-insoluble form likely corresponds to the flagellar cytoskeleton-associated LdMyo13, while the soluble form may act as an actin-based motor within the cell, as supported by in vitro findings [111]. Reverse genetics studies have confirmed that LdMyo13 is essential for flagellum biogenesis and intracellular trafficking in *Leishmania* [113].

## 4. Regulation of Actin Dynamics in *Leishmania*

*Leishmania* spp. harbor only a limited repertoire of canonical actin regulators, yet they display remarkably rapid actin dynamics. Compared with mammalian cells, *Leishmania* actin filaments undergo turnover nearly 20 times faster, depolymerize more efficiently from both ends, and are severed by ADF/cofilin with over 100-fold greater potency [36,73]. These unique properties likely account for the striking absence of long, well-defined actin filaments in intact cells.

**Figure 9:**
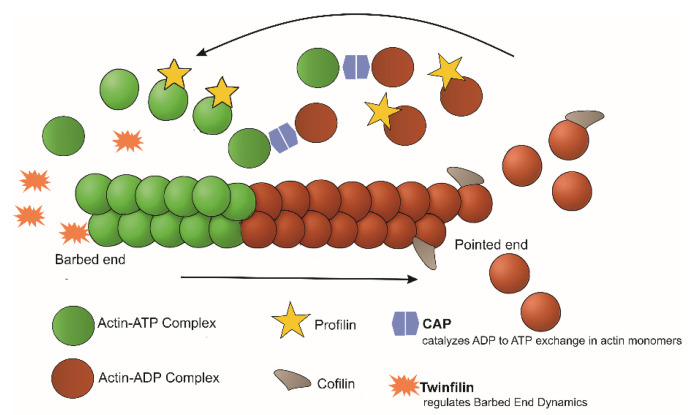
Cartoon depicting the regulation of actin dynamics in *Leishmania*.

Four ABPs, profilin, ADF/cofilin, twinfilin, and CAP, are key regulators of actin dynamics in *Leishmania* (Figure 9). Profilin promotes filament assembly by binding monomeric actin through a unique WH2-like motif, stabilizing the monomer and accelerating ADP-to-ATP exchange to replenish the pool of ATP-actin [40,52]. ADF/cofilin drives rapid filament turnover by severing filaments with far greater efficiency than its mammalian counterpart [36,73]. Twinfilin regulates barbed-end dynamics by blocking polymerization and actively promoting depolymerization, even in the absence of capping protein [36]. Meanwhile, CAP supports sustained polymerization by catalyzing nucleotide exchange on monomers released from aged filaments, ensuring a continuous supply of ATP-actin [36]. Collectively, the interplay between monomer sequestration and capping (twinfilin), nucleotide exchange (profilin and CAP), and highly efficient filament severing (ADF/cofilin) underpins the exceptionally dynamic actin cytoskeleton of *Leishmania*, enabling critical processes such as morphogenesis, motility, endocytosis, and cytokinesis.

In contrast, the mechanisms underlying actin nucleation remain poorly understood. The precise composition and regulation of the ARP2/3 complex are not fully resolved, and *Leishmania* formins have yet to be structurally and functionally characterized. Moreover, profilin has been shown to inhibit formin-mediated nucleation and filament elongation [40], further complicating the picture. These gaps highlight the need for deeper investigation into the molecular mechanisms that govern actin filament nucleation.

Unlike higher eukaryotes, which rely on Rho-family GTPases and kinase/phosphatase pathways to regulate actin remodeling [114,115], *Leishmania* lack canonical Rho/Rac GTPases or possess highly divergent homologs [29]. Instead, they retain primarily RAB and ARL GTPases linked to vesicular trafficking rather than direct actin control [116,117,118], reflecting a fundamental evolutionary divergence.

Despite the absence of classical actin-regulatory GTPases, *Leishmania* may utilize alternative pathways to regulate actin-dependent processes such as endocytosis and cytokinesis. These include calcium signaling, which influences actin polymerization, motility, and cell shape [119,120,121]; cAMP signaling, associated with stress response, differentiation, and cytoskeletal reorganization [122,123]; Lipid signaling, involving PIP2 and other phospholipids that modulate interactions between the plasma membrane and actin cytoskeleton during endocytosis and cell division [124,125,126].

Overall, *Leishmania* employ a distinctive, highly dynamic actin regulatory system that departs sharply from canonical eukaryotic paradigms, an evolutionary innovation that merits deeper mechanistic dissection.

## 5. Potential Roles of Actin and Actin-Binding Proteins During Differentiation and Development of *Leishmania* Parasites

As it is quite evident from the preceding sections, actin remodeling plays an essential role in multiple cellular processes of *Leishmania* parasites. Crucial among them are its role in regulating the cell morphogenesis [73], motility [73,113], endocytosis [40,52], intracellular vesicle trafficking [52,78,113] and cytokinesis [55,78,90,97]. Actin remodeling likely plays an indispensable role in various stages of *Leishmania* parasite development and differentiation [127,128,129,130]. For instance, actin remodeling in cooperation with pellicular microtubules may be responsible for drastic changes in cell morphology [73] during transitions between the promastigote (insect vector) and amastigote (mammalian host) stages of *Leishmania* [128]. Furthermore, actin remodeling is critical for promastigote’s flagellar motility [73,113], enabling the parasite to escape the blood bolus, migrate anteriorly within the sand fly, undergo transient attachment and detachment, reach the proboscis, and be efficiently transmitted during feeding, processes essential for its survival and transmission [127,130].

Beyond cell shape and motility regulation, actin remodeling is pivotal for the parasite’s endocytic and exocytic activities [40,52,78,113]. These processes occur exclusively at the flagellar pocket, which is a microtubule-free invagination of the plasma membrane from where the flagellum emerges. Both the promastigote and amastigote stages of *Leishmania* rely heavily on the flagellar pocket for the uptake of essential nutrients, host defense evasion and virulence factors required during parasite’s invasion and survival within the host cells as well as for the secretion of metabolic waste [75,76].

Actin and its regulators, particularly ADF/cofilin and myosin 13, are essential for the assembly and motility of the *Leishmania* flagellum as well as for maintaining structural integrity of the flagellar pocket. The flagellum consists of two main parts: the axoneme and the paraflagellar rod (PFR). The axoneme features a core “9 + 2” microtubule structure that drives flagellar beating [131], while the PFR, primarily composed of PFR1 and PFR2 proteins, runs parallel to the axoneme beneath the membrane and is involved in motility and waveform generation [132]. It has been reported that myosin 13 not only associates with the PFR but is also concentrated at the base and tip of the flagellum [109]. Depleting its intracellular pool results in the loss of PFR, shortened flagellar length, impaired motility, and an expanded flagellar pocket [113]. These features are fully restored by replenishing cellular levels of myosin 13 [113]. Similarly, actin, which concentrates around the base region and tip of the flagellum and along with ADF/cofilin in the flagellar pocket [32,73], is crucial for flagellar integrity. Depleting ADF/cofilin leads to the loss of PFR, reduced flagellar length, impaired motility, and an enlarged flagellar pocket [73], effects that are reversed by restoring ADF/cofilin levels [73]. Collectively, these findings clearly indicate that actin dynamics, together with myosin 13, play a vital role in the assembly, functions, and organization of the flagellum and flagellar pocket.

Further, actin and actin-binding proteins critically regulate multiple stages of the *Leishmania* cell division cycle [133,134]. While profilin influences gene transcription and maintains mitotic spindle orientation [55], twinfilin governs DNA synthesis and mitotic spindle elongation [97]. Additionally, ADF/cofilin-mediated actin dynamics is essential during early cell division phases, which include basal body and kinetoplast separation, cleavage furrow progression, and flagellar pocket division [78]. Besides this, coronin remodels microtubules within the subpellicular corset during *Leishmania* cytokinesis [90].

Profilin, a crucial regulator of actin dynamics in *Leishmania* [40,52], not only binds to its primary ligands such as actin, PLP motif-containing proteins, and phosphoinositides [52], but it also interacts with various cytoplasmic proteins [55,56]. These include mitochondrial outer membrane protein porin, mRNA processing and translation initiation proteins like eIF4A1, and calpain-like cysteine proteases [55,56], indicating its potential role in regulating mitochondrial functions, gene expression, parasite virulence, and possibly immune evasion. The interactions of profilin with porin and calpain-like cysteine proteases seem to be actin-dependent, as profilin lacking the actin-binding domain does not interact with these proteins [56], highlighting the importance of actin dynamics in the regulation of mitochondrial functions in *Leishmania*.

In addition to profilin, the actin-like protein ALP3 (formerly Arp1) and the actin-related protein ARP4 serve as key regulators of mitochondrial function in *Leishmania* [135,136]. ALP3 is indispensable, localizing predominantly to the mitochondrion where it maintains membrane potential and fuels ATP synthesis, processes critical for parasite survival within host macrophages [135]. In contrast, ARP4 acts as a negative regulator of Ca^2+^ homeostasis, underscoring the finely tuned control of mitochondrial dynamics that underlies *Leishmania* pathogenicity [136].

## 6. *Leishmania* Actin and Actin-Binding Proteins as Potential Drug Targets

*Leishmaniasis* is a neglected tropical disease that primarily affects impoverished populations in the Old World. It presents in three clinical forms: cutaneous (CL), mucocutaneous (MCL), and visceral *Leishmaniasis* (VL), also known as kala-azar. Among these, CL is the most common, while VL is the most severe and potentially fatal if left untreated. Despite being recognized for decades, effective treatment options remain limited due to several challenges. Current therapies are often associated with high toxicity, substantial costs, prolonged treatment durations, and the emergence of drug resistance [137,138]. These limitations highlight the urgent need for safer, more affordable, and more effective treatments to combat this persistent and debilitating disease. In response to this need, current research has increasingly focused on identifying novel therapeutic targets that are essential for parasite survival yet remain unexploited. One promising direction involves targeting actin and actin-binding proteins (ABPs) in *Leishmania*. As highlighted in the preceding sections, these proteins play key role in several essential processes that are vital not only for parasites’ survival but also for their development and differentiation. Disrupting their functions using small-molecule inhibitors would impair parasite viability and pave the way for the development of new, targeted anti-*Leishmanial* therapies.

Actin and its associated proteins are fundamental to the physiological functions of all eukaryotes, including humans. Their dysregulation has been linked to a wide range of human diseases, including cancer [139,140], cardiomyopathy [141], and defects in skeletal myogenesis [142]. This highlights actin and its regulators as compelling targets for therapeutic intervention. However, despite extensive efforts, no safe and effective agents capable of directly modulating actin polymerization have yet been identified [143]. In contrast, significant progress has been made in developing safe and potent inhibitors of the myosin A motor protein (PfMyoA, PfMyoA ATPase and TgMyoA), offering promising therapeutic avenues for combating infections caused by *Plasmodium falciparum* and *Toxoplasma gondii* [144,145,146].

*Leishmania* actin and its actin-binding proteins are likely critical for parasite differentiation (promastigote to amastigote or vice versa), survival, and infectivity. Both life stages rely on endocytosis to obtain nutrients and essential factors from the extracellular environment; disrupting this pathway would therefore undermine parasite viability. Key drivers of endocytosis in *Leishmania* include actin, profilin [39,52], and ADF/cofilin [36,73]. Because these proteins are structurally distinct from their mammalian counterparts, they represent attractive, selective targets for therapeutic intervention.

*Leishmania* profilin not only promotes endocytosis but also contributes to cell-cycle control and possibly mitochondrial function. Unlike canonical eukaryotic profilins, it contains a unique 20–amino-acid helical insertion that resembles a WH2-like motif and mediates actin binding [39]. Small molecules that block this interaction could selectively impair endocytosis, disrupt cytokinesis, and compromise mitochondrial activities, collectively reducing parasite fitness.

*Leishmania* ADF/cofilin retains an overall conserved fold, but its C-terminal region diverges from mammalian cofilins [36]. The parasite and host proteins also employ distinct actin-binding mechanisms [36], providing an opportunity to develop inhibitors that selectively interfere with the parasite’s actin-severing or depolymerizing activities.

Myosin 13 is a trypanosomatid-specific motor with a high druggability score and a distinctive *Leishmania*-specific tail domain [109,110]. It exists in two states: an inactive, dimeric form and an active, monomeric motor. Calmodulin binding converts the protein from the inactive dimer into the active monomer required for actin-dependent vesicle trafficking and flagellar assembly [111,113]. Because the calmodulin-binding site differs from that of other myosins [111], selectively targeting this interaction could block myosin 13 activation and impair essential intracellular transport processes.

Actin itself is central to cytoskeletal function. *Leishmania* actin forms shorter, less-stable filaments and displays distinct polymerization kinetics compared with mammalian actin [33,36]. Divergence in critical regions, such as the DNase I–binding loop (D-loop) and the hydrophobic plug (H-plug), which mediate protomer interfaces, suggests that small molecules targeting these surfaces could selectively disrupt parasite filament assembly [33,36]. However, designing such inhibitors is technically challenging and requires careful optimization to minimize host off-target effects.

Coronin regulates actin dynamics, microtubule remodeling during cytokinesis, and phagosome formation in *Leishmania* [39,90]. The parasite coronin differs structurally from mammalian coronins, including a unique region, a divergent actin-binding site, and an altered leucine-zipper motif. These differences make coronin a promising selective target for small-molecule inhibition of actin-dependent processes. Coronin also interacts with kinesin K39 and contributes to microtubule remodeling, but the precise K39-binding site and the role of actin in this interaction remain to be defined before coronin can be fully exploited therapeutically.

Twinfilin in *Leishmania* shows an unusual subcellular distribution: it is largely nucleolar and, during cell division, relocates to the nucleus where it supports spindle elongation and DNA synthesis. Twinfilin also participates in actin cytoskeleton remodeling [36]. To evaluate its druggability, we need detailed insights into the motifs that mediate nucleolar localization and the molecular mechanisms by which twinfilin contributes to spindle dynamics and DNA replication.

Collectively, the divergence of *Leishmania* actin and ABPs from their mammalian counterparts presents multiple opportunities for selective therapeutic targeting. Priorities for drug discovery should include: (1) profiling parasite-specific actin–ABP interfaces (e.g., profilin WH2-like insertion, cofilin C-terminus, myosin 13 calmodulin site, coronin actin-binding site); (2) developing small molecules that selectively perturb these interactions; and (3) rigorously testing host selectivity to minimize off-target toxicity.

## 7. Conclusions and Future Directions

Emerging evidence underscores that, beyond their well-established microtubule networks, trypanosomatid parasites, particularly *Leishmania*, rely heavily on actin and its associated proteins to regulate a broad spectrum of cellular processes. While essential functions such as morphogenesis and cell division likely depend on coordinated interactions between actin and microtubule-associated proteins, processes like endocytosis and intracellular trafficking are governed predominantly by the actin cytoskeleton and its regulatory components. Actin and its partners also contribute to the assembly of the *Leishmania* flagellum, a motility structure critical for parasite survival and infectivity.

Several compelling observations highlight the need for deeper investigation. Actin has been detected in the kinetoplast, where it colocalizes with kinetoplast DNA (kDNA) [32,34]; in vitro studies further suggest a role in kDNA remodeling [34], though this remains to be validated in vivo. Profilin has also been observed within the kinetoplast [72], but its function in this organelle is still unknown. Additionally, actin, profilin, and the actin-related protein Arp6 have been identified in the nucleus [32,52,147], yet their precise nuclear roles remain elusive. Similarly, the structures and biological functions of the two formin proteins encoded in the *Leishmania* genome are poorly characterized, as are the contributions of twinfilin and CAP to parasite differentiation, physiology, and host–pathogen interactions.

Critically, all existing studies on actin and its associated proteins in *Leishmania* have focused exclusively on the promastigote stage. To date, no work has examined their roles during the amastigote stage, when the parasite resides and replicates within the hostile environment of host macrophages. Given the profound physiological differences between the two life stages, it is highly probable that these proteins exhibit stage-specific functions, as has already been reported in the related parasite Trypanosoma brucei [148].

There is thus an urgent need to develop tools and models that enable functional characterization of actin, actin-binding, actin-like, and actin-related proteins throughout the parasite’s developmental cycle, particularly during intracellular differentiation and survival. Unraveling these mechanisms will not only advance our understanding of parasite biology but may also reveal novel molecular targets for therapeutic intervention.

## Figures and Tables

**Table 1:** Sequence comparison of *Leishmania* actin against human cytosolic actin.

Species	% Identity	% Similarity
*L. major*	69.5	86.5
*L. donovani*	69.8	86.5
*L. infantum*	70.0	86.5
*L. chagasi*	70.0	86.5
*L. braziliensis*	70.0	85.9
*L. mexicana*	70.0	86.5

All sequences have ~70% identity, ~86% similarity and 0.8% indels.

**Table 2:** Actin-Binding Proteins in Higher Eukaryotes (Adapted from references [12,13] of the manuscript).

Category	Protein(s)	Function
1. Actin Nucleating Proteins	ARP2/3 Complex	Promotes actin nucleation and initiates formation of branched filaments by binding to sides of existing filaments. Activated by WASP/WAVE proteins.
	Formins (mDia1, mDia2, DAAM1)	Promote nucleation and elongation of linear actin filaments by binding to the barbed end.
2. Polymerization/Depolymerization Promoters	Profilin	Sequesters ADP-actin to prevent uncontrolled polymerization; promotes ATP exchange and delivers ATP-actin to barbed ends.
	Thymosin-β4	Sequesters G-actin, preventing polymerization.
	Cofilin/ADF	Binds ADP-G-actin and filaments; promotes severing and depolymerization.
	Aip1	Enhances cofilin-mediated actin filament severing.
	Ena/VASP	Promotes elongation by protecting barbed ends from capping.
	Twinfilin	Prevents filament assembly at barbed end while allowing disassembly.
	Cyclase-Associated Protein (CAP/Srv2)	Promotes pointed-end depolymerization of cofilin-decorated filaments; regulates monomer pool.
	Gelsolin	Caps, severs, and nucleates filaments in a calcium-dependent manner.
3. Actin Capping Proteins	Cap Z	Caps barbed ends, preventing further polymerization.
	Tropomodulin	Caps pointed ends of actin filaments.
4.Crosslinking and Bundling Proteins	α-Actinin	Crosslinks actin filaments into bundles or networks.
	Fimbrin/Plastin	Bundles actin filaments into tight parallel arrays.
	Filamin	Crosslinks actin filaments into orthogonal networks.
	Spectrin	Links actin filaments to the plasma membrane; forms part of the cortical cytoskeleton.
5. Actin-Membrane Linkers	Ezrin/Radixin/Moesin (ERM)	Link actin filaments to the plasma membrane.
	Talin	Links actin filaments to integrins at focal adhesions.
	Vinculin	Stabilizes actin–membrane interactions at focal adhesions.
6. Other Actin-Associated Proteins	Myosin Motors	Interact with actin filaments to generate contractile forces.
	Tropomyosin	Stabilizes actin filaments and regulates their interaction with other proteins.
	Coronin	Binds to actin filaments and stabilizes them.
